# Laser hemorrhoidoplasty vs. rubber band ligation: a randomized trial comparing 2 mini-invasive treatment for grade II hemorrhoids

**DOI:** 10.1186/s12893-024-02425-z

**Published:** 2024-05-27

**Authors:** Lei Jin, Kaijian Qin, Renjie Wu, Haojie Yang, Can Cui, Zhenyi Wang, Jiong Wu

**Affiliations:** 1 Department of Coloproctology, Yueyang Hospital of Integrated Traditional Chinese and Western Medicine, Shanghai University of Traditional Chinese Medicine, Shanghai, 200437 China; 2 Department of Coloproctology, Xiangshan TCM Hospital, Huangpu District, Shanghai, China

**Keywords:** Laser hemorrhoidoplasty, Hemorrhoid, Minimally invasive, Rubber band ligation, Postoperative pain

## Abstract

**Purpose:**

As a minimally invasive procedure, laser hemorrhoidoplasty (LHP) can not only relieve the symptoms of hemorrhoids, but also protect the anal cushion structure. This study aimed to investigate the clinical efficacy of LHP in the treatment of grade II hemorrhoids.

**Methods:**

A total of 70 patients with grade II hemorrhoids were randomly assigned to receive LHP or Rubber Band Ligation (RBL) (*n* = 35 per group) in 2019 from a single center. The postoperative pain, bleeding, feeling of anal distension(local falling, swelling, foreign body sensation, stool) and postoperative recurrence rate were compared between the two groups.

**Results:**

The postoperative pain, bleeding, and feeling of anal distension in the LHP group were improved significantly as compared with the RBL group within 2 weeks after surgery (*P* < 0.01). Both methods can relieve the symptoms of grade II hemorrhoids. There was no difference in the recurrence rate between the two groups at 1 year after surgery (*P* > 0.05). The patients in LHP group took less time to return to normal activities (*P* < 0.001).

**Conclusions:**

As a minimally invasive treatment, LHP is easy and not traumatic and results in mild postoperative pain and few complications. It is an ideal choice for grade II hemorrhoids.

**Supplementary Information:**

The online version contains supplementary material available at 10.1186/s12893-024-02425-z.

## Introduction

Hemorrhoids are one of the most common anal diseases in clinical practice. In China, the incidence of anorectal diseases in adults is 50.1%, and hemorrhoids account for 98.1% of total anorectal diseases [1]. It has been reported that over 50% of people present at least one episode of symptomatic hemorrhoids during their life [2].If conservative treatment cannot work, surgical management is required. Excision hemorrhoidectomy is the most effective treatment for hemorrhoides with the lowest recurrence rate compared to other methods [3].But postoperative pain and complications are still unavoidable problems. Therefore, for the fear of postoperative pain and complications, mildly symptomatic patients often hesitate and delay undergoing surgical treatment for this benign disease [4]. In addition, it is not properly to treat low-grade hemorrhoidal patients using the same surgical techniques [5]. Of course, for bleeding grade II hemorrhoids, other minimally invasive surgical therapies can also be applied to patients who have failed conservative treatment, such as Transanal hemorrhoidal dearterialization(THD) [6], and are safe with minimal complications such as pain and bleeding [7]. But sometimes this requires the assistance of an ultrasound specialist.

Rubber Band Ligation (RBL) has been generally recognized as a safe and effective means of treating grade II hemorrhoids and partial grade III hemorrhoids. But the most common complication of RBL is pain and rectal discomfort [8]. As a non-excisional laser therapy, Laser Hemorrhoidoplasty (LHP) was first described in 2007 by Karahaliloglu et al. [9]. With the LHP-technique, a laser fibre is to be inserted into the hemorrhoid and laser energy is applied. Absorption of laser energy by the hemorrhoid tissue leads to destruction of the hemorrhoid vessels, followed by submucosal fibrotic shrinking and reduction of total hemorrhoid tissue. In principle there is adapted individual shrinkage of every single node and no excision of the hemorrhoid at all. The aim is a protection of the anal cushion, the hemorrhoid artery is obliterated, improving bleeding symptoms. In case of prolapse the shrinking of the hemorrhoidal mass and fibrotic reconstruction will lead to reduce the prolapse problem.

As a team that used laser technology earlier in China, we conducted a randomized controlled study on LHP and RBL to compare the performance of these two minimally invasive procedures in terms of postoperative pain, complications and mid-term recurrence, aiming to evaluate the clinical efficacy of LHP for grade II hemorrhoids. We are going to explore whether the simpler, less painful and faster recovery surgical methods are suitable for our domestic situation. Here, we report our initial experience gained from developing this minimally invasive technology.

## Materials and methods

This is a randomized (1:1), single-center prospective study. This study was performed in the Anorectal Department, Yueyang Hospital of Integrative Traditional Chinese and Western Medicine, Shanghai University of Traditional Chinese Medicine, Shanghai, China. Yueyang Hospital is a large tertiary university hospital. All patients signed the informed consent form for participation in the study in addition to the consent form for the operation. Patients were randomly assigned through a computer-generated randomization list to receive LHP or RBL (*n* = 35 per group). This study was approved by the Ethics Committee of Yueyang Hospital (No. 2019-043).

### Inclusion and exclusion criteria

Inclusion criteria were patients (aged 18–65 years) with symptomatic degree II hemorrhoids, who failed with conservative treatment previously, were consented to participate. Exclusion criteria were hemorrhoids of degree I III and circumferential hemorrhoids, pregnancy, menstruation at the time of surgery, patients with other anorectal diseases (fistula, abscess, rectal carcinoma, inflammatory bowel disease, etc.), patients who underwent anal operations within 6 months and patients with severe mental illness or severe acute infectious diseases.

### Operative procedure

Before surgery, all patients underwent laboratory tests, chest X-ray and ECG examination. Preoperatively, every one took oral laxatives for colonoscopy to exclude the presence of inflammatory bowel disease and neoplasia. No need for preventive antibiotic before surgery.

All surgical procedures were performed by the same surgeon (JW), who is experienced in coloproctological surgery, assisted by a skilled collaborative team.

Lateral position and general anesthesia for all patients during surgery.

### The procedures for LHP

**Fig. 1:**
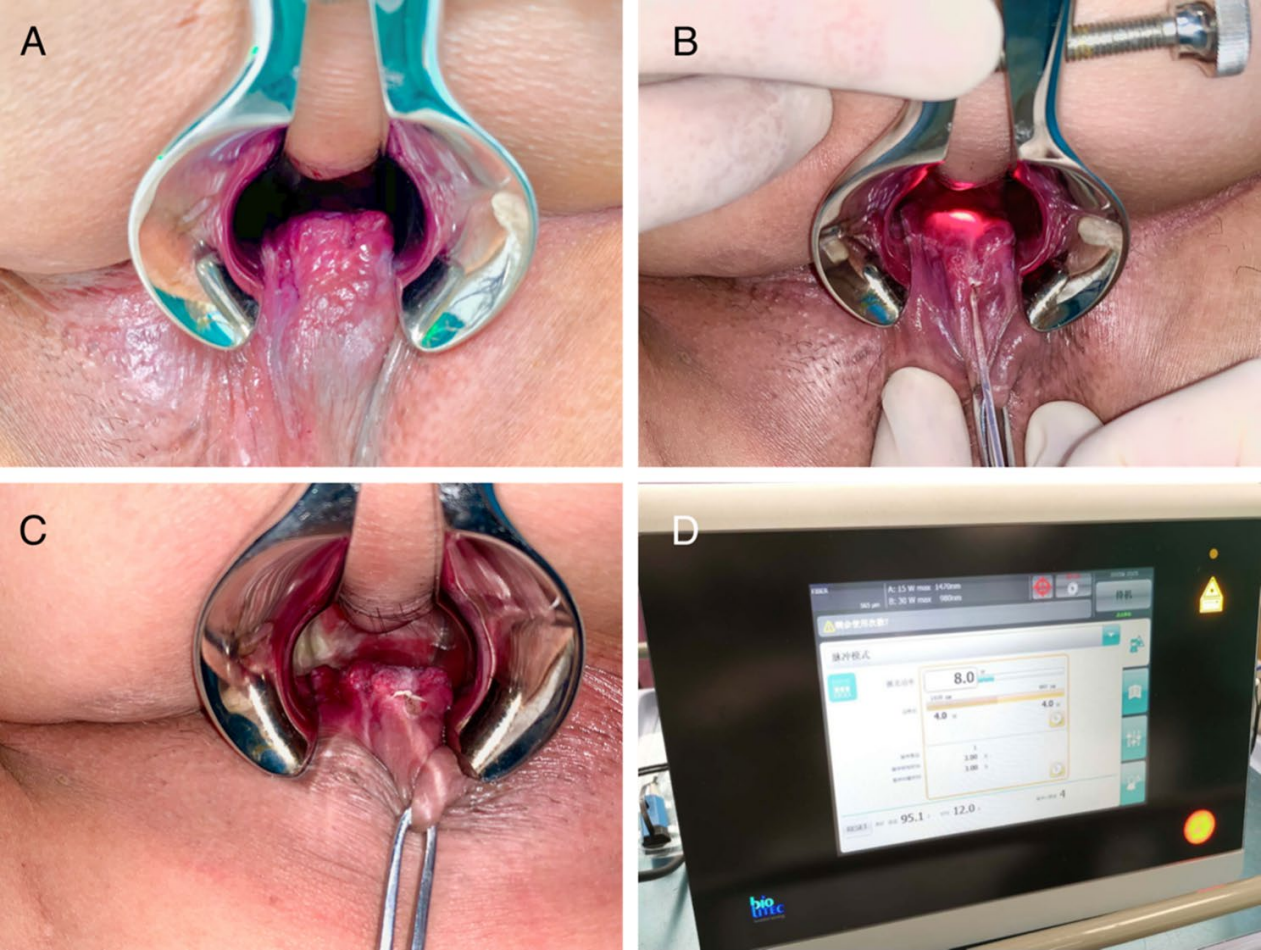
A: Preoperative identification of the hemorrhoids. B: Positioning according to the guiding light C: Postoperative image. D: Diode laser generator(Leonardo® DUAL 45 CeramOptec GmbH of Biolitec)

The hemorrhoids were checked directly under a half-anoscope and the area to be treated was selected (Fig. 1-A). A 2–3 mm skin microincision was made at the anal verge of each pile. First, the laser fiber was introduced through the skin microincision until the root of the internal hemorrhoids where is above the dentate line and positioned according to the guiding light of the laser fiber (Fig. 1-B). The laser fiber was placed between the mucosa and the internal sphincter to avoid damaging them. After surgery, Partial atrophy of hemorrhoids(Fig. 1-C). The machine was set with an 8 W 980 + 1470 nm (50%/50%) Diode laser (Leonardo ® DUAL 45 CeramOptec GmbH of Biolitec® AG, Germany) (Fig. 1-D). Each pulse lasted 3 s. For each hemorrhoid area, the laser energy release points are distributed in a fan-shaped manner and generally six points were burned to ensure that the entire hemorrhoid area was treated. The procedure was repeated in other obvious hemorrhoid area. Cool surface of every treated pile directly after laser use by ice cube.

### The procedures for RBL

The hemorrhoids were directly localized under the oblique transparent anal endoscope and graded. The more prominent hemorrhoids are generally on right anterior, right posterior, and left lateral position. If obvious hemorrhoid was seen in other area, it would also be ligated. Keep the mucosa intact between the adjacent two area. The instrument with disposable elastic band (Well, Wuhan Medical Technology Co., Ltd., B-6 H) was used for the hemorrhoid ligation. The mucosa and submucosal tissues were suctioned into the instrument, but the suction of the muscular tissues should be avoided. The negative pressure was − 0.1 MPa [10]. Put one rubber band on the base of each hemorrhoid. After ligation, anus was examined for bleeding and stenosis and ensured the hemorrhoids retracted well.

Compound Carraghenates suppositories were used in both groups postoperatively, no antibiotics were need.

Postoperative anal pain was evaluated with the visual analog scale (VAS) within 24 h and at 1 day, 3 days, 7 days and 14 days after surgery. Postoperative bleeding and feeling of anal distension were evaluated at 1, 3 and 7 days(yes/no). Definition of bleeding: bleeding during defecation, and the bleeding stops at the end of the defecation. Definition of anal distension: local falling, swelling, foreign body sensation, stool. Postoperative recurrence was defined as the presence of bloody stool or hemorrhoid prolapsing, which need medicine to relieve.

Patients were required to be followed up at 7 days,14 days,1 month, and 1year after operation. Postoperative assessment team consisted of 4 independent investigators who had no participated in surgery. During the follow-up, all patients were asked the occurrence of symptoms (pain, bleeding, feeling of anal distension, prolapse) and adverse events via outpatient interview combined telephone or app.

### Statistical analysis

Data with a normal distribution are expressed as mean ± standard deviation (± S), and data with a non-normal distribution are expressed as median and interquartile range. Quantitative variables were compared between two groups using the independent *t* test or the Mann–Whitney U test. The chi-square test was used for the comparison of qualitative variables. The recurrence rate was analyzed by calculating the cumulative incidence. A value of *P* < 0.05 was considered to indicate statistical significance. Statistical analysis was performed using SPSS version 21.0 and GraphPad Prism 6 [10].

## Results

The baseline characteristics of patients in the two groups are shown in Table 1.

**Table 1:** Demographic and clinical characteristics of patients in the two groups

Characteristics	*LHP* (*N* = 35)	*RBL* (*N* = 35)	*P*
Male/female	19/16	18/17	0.81
Age, y	43.28 ± 11.33	44.68 ± 12.14	0.62

*Notes* There were no significant differences in age, gender between the two groups (*P* > 0.05)

### Postoperative outcomes

**Table 2:** Postoperative outcomes

Ending Point	LHP(*n* = 35)	RBL(*n* = 35)	*P*
**Postoperative pain**			
<24 h	2(0–4)	3(2–5)	<0.001
1d	1(0–4)	3(1–4)	<0.001
3d	0(0–3)	2(0–4)	<0.001
7d	0(0–1)	1(0–2)	<0.001
14d	0(0–0)	0(0–1)	<0.01
**Bleeding, n(%)**			
1d	1(2.9%)	14(40%)	<0.001
3d	0(0%)	12(34.3%)	<0.001
7d	0(0%)	20(57.1%)	<0.001
**Feeling of anal distention, n(%)**			
1d	12(34.2%)	34(97.1%)	<0.001
3d	7(20%)	33(94.2%)	<0.001
7d	3(8.5%)	25(71.4%)	<0.001
Return to normal daily activities (± S, day)	3.62 ± 0.91	7.65 ± 1.64	<0.001
Recurrence rate,1year	5.9% (2/35)	11.4% (4/35)	0.41

**Fig. 2:**
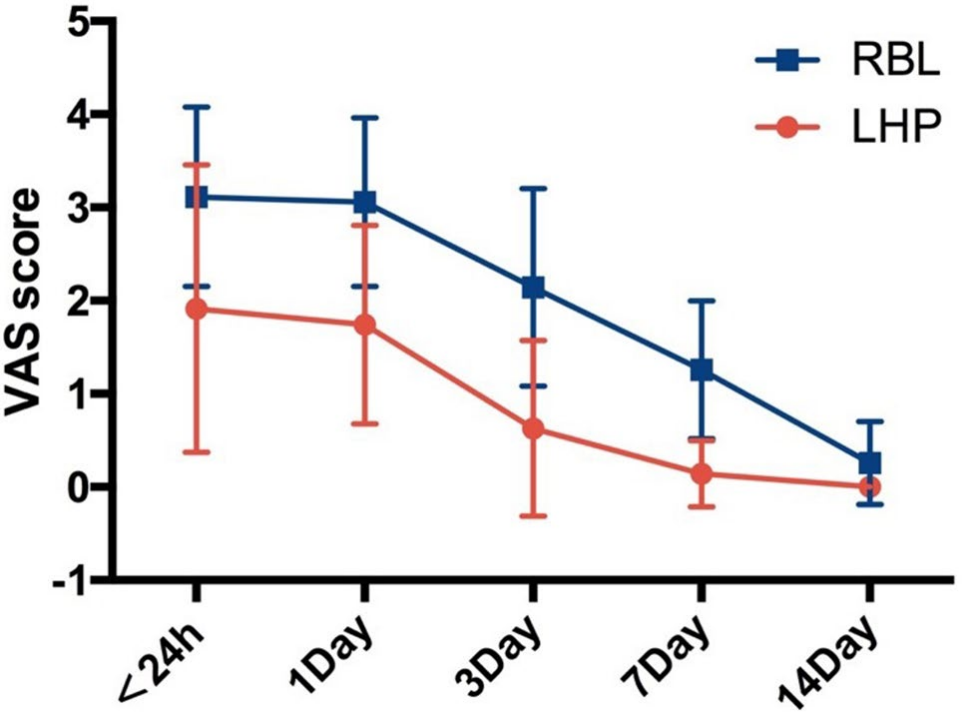
Postoperative pain score

Table 2 summarizes the postoperative outcomes. Within 24 h to 7 days after surgery, postoperative pain was significantly lower after LHP compared with RBL (*P* < 0.001) (Fig. 2). Postoperative bleeding and the feeling of anal distension were markedly better in the LHP group compared to the RBL group (*P* < 0.001). The patients in LHP group took less time to return to normal activities than patients in RBL group (*P* < 0.001). None patient in either group experienced postoperative local infection and incontinence. Mild urinary retention (symptom could be relieved by oral medication) was observed in six patients in the RBL group and no patients in the LHP group.

None was lost to follow-up in the two groups. All patients’ symptoms were relieved after surgery. Within 1 year of follow-up, two patients developed prolapsing hemorrhoid in the LHP group. In the RBL group, four patients developed blood stool (mild bleeding). These 6 patients’ symptom could be relieved by oral and topical medication. The recurrence rate of the two groups had no difference (*P*>0.05).

## Discussion

Since Thomson proposed the concept of “Anal Cushion” [11], people have realized that hemorrhoids in humans are caused by the alteration of anorectal anatomic structures. To protect normal structures, more and more minimally invasive procedures have emerged. These procedures improve symptoms of hemorrhoids by blocking the blood supply while protecting the anal cushion structure. Such as Rubber Band Ligation (RBL)、Doppler-guided hemorrhoidal artery ligation(DGHAL)、Stapled Hemorrhoidopexy(SH) and Laser Hemorrhoidoplasty (LHP). DGHAL can relieve bleeding of grade II or III hemorrhoids effectively instead of improving prolapsing.SH is reserved for circumferential prolapsing hemorrhoids. It has less complications as well as higher degree of patient satisfaction. However, in the longer term, SH was associated with a higher rate of prolapse [12].RBL has been generally recognized as a safe and effective mini-invasive office technique for the treatment of symptomatic early hemorrhoids [13]. Short-term recurrence rates reported in the literature for this procedure range from 12–18% [14]. With the development of instruments, the way of ligation has changed accordingly. But there are some problems, such as sudden shedding bleeding, which is a common problem in resection surgery. Karahaliloglu first used LHP to treat hemorrhoids in 2007.As an emerging non-excisional treatment, a systematic review and meta-analysis has shown that LHP has favorable short-term clinical outcomes in treating grade II/III hemorrhoids compared to traditional surgery, reducing pain and allowing for earlier resumption of work or daily activities [15]. And compared to RBL, LHP has been reported lower postoperative pain than RBL, and recurrence rate was reported to range between 0 and 11.3% after LHP [16].

Our research showed that postoperative pains scores on the VAS were significantly lower after LHP than after RBL during one week after surgery. The result might be attributed to two reasons. On one hand, multiple band ligations (in 3 positions or more) were made in RBL group, which was also mentioned by Giamundo [13].Although the rubber band should be as far away as possible from the dentate line, as a foreign body, which will cause the patient to feel swelling until it sloughs off. It might be responsible for the mild urinary retention reported by 6 cases and anal distention significantly after RBL. On the other hand, that was due to the preservation of mucosal integrity in LHP group. The laser releases energy under the mucosa, it did not destroy the integrity of the mucosa at all. The only damage was a small microincision in the skin of the anal margin for the laser fiber to enter and exit, so the pain after laser surgery was significantly lower than that after RBL.

We were pleased to find that the probability of bleeding after LHP was significantly lower than RBL. Due to the delay bleeding when the banded tissue sloughs off, the proportion of patients with blood stool within one week after RBL was higher than after LHP group, and there was a significant difference between the two groups.

Regarding resolution of symptoms, both methods were satisfactory. On account of light pain and less complications, the patients after LHP could return to normal activities faster than after RBL. After one year of follow-up, the 5.9% recurrence rate in the LHP group is consistent with the results reported by others [4, 9, 17, 18]. Although there was no difference in the recurrence rate between the two groups, it could be seen that LHP was superior to RBL in resolving bleeding and inferior to RBL in improving the symptoms of hemorrhoids prolapse.

A systematic review [16] of seven LHP studies mentioned that resolution of grade II and III hemorrhoids symptoms ranged between 70% and 100% after LHP. It showed lower postoperative pain, but the most commonly reported postoperative complication was bleeding (range 0–64%). In severe cases, sutures were needed to stop bleeding. These research used different wavelengths of lasers, and due to the novelty of technology, the proficiency of surgeons in the use of lasers varies, which can greatly affect postoperative complications. So in the future, LHP will be better and more widely used by surgeons, and its therapeutic effects will continue to improve.

Most of these studies used either 980–1470 nm lasers. Lasers with a wavelength of 980 nm are chosen in most studies. This kind of short-wavelength laser is mainly absorbed by hemoglobin; it damages blood vessel walls through heat release and has a good hemostatic effect. However, its absorption efficiency is low, and the required working energy is high (12–18 W), so damage to the surrounding tissue and postoperative pain are inevitable, and tissue carbonization is obvious. Plapler et al. [16] mentioned that scars were formed because of burn lesions in four patients. They concluded that on the one hand, this is related to the doctor’s experience, and on the other hand, the more energy is applied for too long or too close to the mucosa, the greater is the chance of tissue damage.

The 1470 nm laser is mainly absorbed by water and has relatively little effect on hemoglobin. The heat can be concentrated in a small volume of tissue, causing the necrotic tissue to rapidly decompose and vaporize, which is beneficial to reduce skin paresthesia and local pain [17, 18]. The 1470 nm laser exhibits a high tissue absorption rate, has a low penetration depth and requires only 6–8 W, and hence it can effectively control the tissue damage range and avoid damage to normal tissue. Based on the above reasons, we chose 980 and 1470 nm double-wave treatment, which can not only achieve a good hemostatic effect, less postoperative bleeding, but also requires only 8 W for effective treatment, so damage to normal tissue is avoided.

The following is a summary of our experience with this minimally invasive technology: (1) The path is fan-shaped during laser delivery to ensure that all blood vessels in the hemorrhoids are destroyed. (2) When firing the laser, the laser fiber and the mucosa must not be too close; otherwise the mucosa may be burned. (3) When laser treatment is performed on the area of obvious internal hemorrhoids, it is recommended to treat the external hemorrhoids together to avoid edema after surgery.

The limitations of study was a single-center study and lack of long-term follow-up. Larger samples will provide more reliable data to support clinicians’ surgical decision. We also suggest that for different types of hemorrhoids, LHP can not only be used alone, but also can be combined with other technologies. How to reduce postoperative pain and complications while improving efficiency and bringing more benefits to patients is what we need to think about in the future.

## Conclusion

Both LHP and RBL are safe and effective treatment for grade II hemorrhoids. These techniques are both mini-invasive. LHP is superior in postoperative pain and complications. The most important advantage of LHP is that it enables patients to quickly return to normal life, with very little impact on work and study. As a non-excisional treatment, LHP is an ideal choice for grade II hemorrhoids. This technique opens new possibilities for the surgical treatment of hemorrhoidal disease.

### Electronic supplementary material

Below is the link to the electronic supplementary material.

Supplementary Material 1

Supplementary Material 2

## Data Availability

As a minimally invasive treatment, LHP is easy and not traumatic and results in mild postoperative pain and few complications. It is an ideal choice for grade II hemorrhoids and it is available from corresponding author upon reasonable request.
