# Supplementary material for: Laser hemorrhoidoplasty vs. rubber band ligation: a randomized trial comparing 2 mini-invasive treatment for grade II hemorrhoids
Source: BMC Surg. 2024 May 27;24:164. doi: 10.1186/s12893-024-02425-z (PMC11129420; doi:10.1186/s12893-024-02425-z)
Supplement: Supplementary file 2 — Supplementary Material 2 [file 12893_2024_2425_MOESM2_ESM.docx]

Supplementary table S1

This table includes the basic information and postoperative pain scores of 35 patients who used Laser Hemorrhoidoplasty.Postoperative anal pain was evaluated with the visual analog scale (VAS) within 24 h and at 1 day, 3 days, 7 days and 14 days after surgery. The time required to restore normal daily activities in the end.

| Number | Name | Sex | Age | Postoperative pain（＜24h） | Postoperative pain（1d） | Postoperative pain（3d） | Postoperative pain（7d） | Postoperative pain（14d） | Return to normal daily activities  （day） |
| --- | --- | --- | --- | --- | --- | --- | --- | --- | --- |
| 1 | Wu QW | M | 36 | 2 | 3 | 0 | 0 | 0 | 3 |
| 2 | Li RY | F | 30 | 0 | 0 | 0 | 0 | 0 | 5 |
| 4 | Wang LH | M | 37 | 0 | 2 | 0 | 0 | 0 | 3 |
| 9 | Ling ZY | M | 50 | 0 | 1 | 3 | 0 | 0 | 3 |
| 10 | Li WJ | M | 32 | 4 | 4 | 0 | 0 | 0 | 5 |
| 13 | Jia YA | M | 33 | 1 | 1 | 0 | 0 | 0 | 4 |
| 14 | Hua T | M | 35 | 1 | 1 | 0 | 0 | 0 | 5 |
| 15 | Ma JG | M | 60 | 3 | 1 | 0 | 0 | 0 | 3 |
| 16 | Liu YD | F | 47 | 1 | 1 | 0 | 0 | 0 | 3 |
| 18 | Liu BQ | M | 60 | 3 | 3 | 1 | 0 | 0 | 4 |
| 19 | Lu B | M | 37 | 4 | 2 | 1 | 0 | 0 | 3 |
| 20 | Liu Y | M | 41 | 1 | 1 | 0 | 0 | 0 | 3 |
| 21 | Pu CM | F | 31 | 3 | 3 | 0 | 0 | 0 | 4 |
| 23 | Wang FP | F | 29 | 1 | 1 | 0 | 0 | 0 | 4 |
| 26 | Liu BH | M | 50 | 0 | 0 | 0 | 0 | 0 | 3 |
| 30 | Xu L | F | 44 | 3 | 2 | 0 | 0 | 0 | 2 |
| 33 | Lu X | F | 44 | 4 | 3 | 3 | 1 | 0 | 4 |
| 35 | Liu HX | M | 32 | 3 | 3 | 2 | 1 | 0 | 3 |
| 38 | Lu LM | M | 65 | 0 | 0 | 0 | 0 | 0 | 3 |
| 39 | Huang RM | M | 63 | 1 | 1 | 0 | 0 | 0 | 4 |
| 41 | Zhang YK | M | 54 | 4 | 2 | 2 | 1 | 0 | 3 |
| 42 | Pu YF | M | 48 | 3 | 3 | 2 | 0 | 0 | 3 |
| 44 | Yu YP | F | 64 | 3 | 2 | 0 | 0 | 0 | 3 |
| 48 | Zheng JX | F | 36 | 4 | 2 | 1 | 1 | 0 | 3 |
| 52 | Huang HJ | M | 37 | 3 | 3 | 2 | 1 | 0 | 2 |
| 55 | Yang G | M | 41 | 0 | 1 | 0 | 0 | 0 | 4 |
| 56 | Dai X | M | 29 | 4 | 3 | 1 | 0 | 0 | 5 |
| 57 | Huang WT | F | 55 | 1 | 1 | 0 | 0 | 0 | 5 |
| 60 | Gu YQ | F | 39 | 0 | 1 | 0 | 0 | 0 | 3 |
| 61 | Yang QX | F | 38 | 2 | 1 | 1 | 0 | 0 | 5 |
| 65 | Wu LL | F | 46 | 0 | 1 | 0 | 0 | 0 | 4 |
| 66 | Xiang YX | F | 24 | 0 | 1 | 0 | 0 | 0 | 5 |
| 68 | Chen L | F | 60 | 3 | 3 | 1 | 0 | 0 | 3 |
| 69 | Chai LN | F | 40 | 4 | 3 | 2 | 0 | 0 | 3 |
| 70 | Sun YJ | F | 48 | 1 | 1 | 0 | 0 | 0 | 5 |

Supplementary table S2

This table includes the basic information and postoperative pain scores of 35 patients who used Rubber Band Ligation. Postoperative anal pain was evaluated with the visual analog scale (VAS) within 24 h and at 1 day, 3 days, 7 days and 14 days after surgery. The time required to restore normal daily activities in the end.

| Number | Name | Sex | Age | Postoperative pain（＜24h） | Postoperative pain（1d） | Postoperative pain（3d） | Postoperative pain（7d） | Postoperative pain（14d） | Return to normal daily activities  （day） |
| --- | --- | --- | --- | --- | --- | --- | --- | --- | --- |
| 3 | Yang GY | F | 61 | 4 | 3 | 2 | 2 | 1 | 9 |
| 5 | Zhao GL | M | 63 | 4 | 4 | 3 | 2 | 0 | 5 |
| 6 | Chen Y | F | 30 | 3 | 3 | 3 | 2 | 1 | 7 |
| 7 | Han J | F | 36 | 2 | 2 | 2 | 1 | 1 | 8 |
| 8 | Niu KZ | M | 40 | 2 | 2 | 1 | 1 | 0 | 5 |
| 11 | Zhou JX | M | 64 | 2 | 1 | 0 | 0 | 0 | 6 |
| 12 | Chen W | M | 37 | 4 | 2 | 2 | 1 | 1 | 6 |
| 17 | Zhou HX | M | 46 | 3 | 3 | 1 | 1 | 0 | 8 |
| 22 | Feng QB | M | 38 | 4 | 2 | 0 | 0 | 0 | 9 |
| 24 | Hou YQ | F | 61 | 4 | 4 | 3 | 2 | 1 | 8 |
| 25 | Teng J | F | 30 | 3 | 3 | 1 | 1 | 0 | 9 |
| 27 | Li Q | F | 30 | 5 | 4 | 3 | 2 | 0 | 8 |
| 28 | Qian TC | M | 42 | 4 | 4 | 3 | 2 | 1 | 6 |
| 29 | Fan LL | F | 32 | 2 | 4 | 2 | 1 | 0 | 7 |
| 31 | OU Yang | F | 28 | 3 | 3 | 2 | 1 | 0 | 5 |
| 32 | Feng L | M | 36 | 2 | 2 | 1 | 1 | 0 | 9 |
| 34 | Dai Yq | F | 59 | 3 | 3 | 1 | 1 | 1 | 9 |
| 36 | Li AM | F | 59 | 2 | 2 | 1 | 0 | 0 | 7 |
| 37 | Zhou JR | M | 51 | 2 | 2 | 2 | 0 | 0 | 6 |
| 40 | Lu DR | M | 57 | 3 | 4 | 2 | 1 | 1 | 7 |
| 43 | Huang XP | F | 48 | 3 | 3 | 2 | 2 | 0 | 10 |
| 45 | Zhang QY | F | 63 | 4 | 4 | 2 | 1 | 0 | 7 |
| 46 | Jiang JJ | M | 49 | 3 | 4 | 4 | 2 | 0 | 10 |
| 47 | Wang M | F | 29 | 5 | 4 | 2 | 2 | 0 | 11 |
| 49 | Zhu JL | F | 37 | 2 | 4 | 4 | 2 | 0 | 8 |
| 50 | Pan CL | M | 44 | 3 | 4 | 3 | 1 | 1 | 7 |
| 51 | Chhen L | M | 43 | 4 | 4 | 4 | 2 | 0 | 7 |
| 53 | Chen HF | F | 62 | 3 | 2 | 2 | 2 | 0 | 6 |
| 54 | Wang Z | M | 25 | 3 | 3 | 2 | 2 | 0 | 9 |
| 58 | Lu L | M | 44 | 2 | 2 | 2 | 1 | 0 | 10 |
| 59 | Liu XL | M | 38 | 3 | 4 | 4 | 0 | 0 | 10 |
| 62 | Fu C | M | 46 | 5 | 4 | 3 | 2 | 0 | 5 |
| 63 | Lu WS | F | 32 | 2 | 3 | 3 | 2 | 0 | 8 |
| 64 | Wu RP | F | 60 | 2 | 2 | 1 | 0 | 0 | 9 |
| 67 | Jiiao J | M | 44 | 4 | 3 | 2 | 1 | 0 | 7 |

Supplementary table S3

This table includes the number of postoperative bleeding, anal distention, urinary retention and recurrence of all patients.

| group | lhp | lhp | rbl | rbl |
| --- | --- | --- | --- | --- |
| ending | no | yes | no | yes |
| bleeding 1day | 24 | 11 | 1 | 34 |
| bleeding 3day | 29 | 6 | 3 | 32 |
| bleeding 7day | 31 | 4 | 23 | 12 |
| anal distention 1day | 23 | 12 | 1 | 34 |
| anal distention 3day | 28 | 7 | 2 | 33 |
| anal distention 7day | 32 | 3 | 10 | 25 |
| urinary retention | 35 | 0 | 29 | 6 |
| recurrence | 33 | 2 | 31 | 4 |
